# Attrition in Web-Based Treatment for Problem Drinkers

**DOI:** 10.2196/jmir.1811

**Published:** 2011-12-27

**Authors:** Marloes G Postel, Hein A de Haan, Elke D ter Huurne, Job van der Palen, Eni S Becker, Cor AJ de Jong

**Affiliations:** ^1^Tactus Addiction TreatmentEnschedeNetherlands; ^2^Nijmegen Institute for Scientist-Practitioners in AddictionNijmegenNetherlands; ^3^Department of EpidemiologyMedisch Spectrum TwenteEnschedeNetherlands; ^4^Department of Research Methodology, Measurement, and Data AnalysisUniversity of TwenteEnschedeNetherlands; ^5^Behavioural Science InstituteRadboud University NijmegenNijmegenNetherlands

**Keywords:** Web-based treatment, online treatment, problem drinking, alcohol, attrition, dropout

## Abstract

**Background:**

Web-based interventions for problem drinking are effective but characterized by high rates of attrition. There is a need to better understand attrition rates in order to improve the completion rates and the success of Web-based treatment programs.

**Objective:**

The objectives of our study were to (1) examine attrition prevalence and pretreatment predictors of attrition in a sample of open-access users of a Web-based program for problem drinkers, and (2) to further explore attrition data from our randomized controlled trial (RCT) of the Web-based program.

**Methods:**

Attrition data from two groups of Dutch-speaking problem drinkers were collected: (1) open-access participants enrolled in the program in 2009 (n = 885), and (2) RCT participants (n = 156). Participants were classified as noncompleters if they did not complete all 12 treatment sessions (9 assignments and 3 assessments). In both samples we assessed prevalence of attrition and pretreatment predictors of treatment completion. Logistic regression analysis was used to explore predictors of treatment completion. In the RCT sample, we additionally measured reasons for noncompletion and participants’ suggestions to enhance treatment adherence. The qualitative data were analyzed using thematic analysis.

**Results:**

The open-access and RCT group differed significantly in the percentage of treatment completers (273/780, 35.0% vs 65/144, 45%, χ^2^_1_ = 5.4, *P* = .02). Logistic regression analysis revealed a significant contribution of treatment readiness, gender, education level, age, baseline alcohol consumption, and readiness to change to predict treatment completion. The key reasons for noncompletion were personal reasons, dissatisfaction with the intervention, and satisfaction with their own improvement. The main suggestions for boosting strategies involved email notification and more flexibility in the intervention.

**Conclusions:**

The challenge of Web-based alcohol treatment programs no longer seems to be their effectiveness but keeping participants involved until the end of the treatment program. Further research should investigate whether the suggested strategies to improve adherence decrease attrition rates in Web-based interventions. If we can succeed in improving attrition rates, the success of Web-based alcohol interventions will also improve and, as a consequence, their public health impact will increase.

**Trial:**

International Standard Randomized Controlled Trial Number (ISRCTN): 39104853; http://www.controlled-trials.com/ISRCTN39104853 (Archived by WebCite at http://www.webcitation.org/63IKDul1T)

## Introduction

Web-based interventions for problem drinkers improve the availability of alcohol treatment services and reach a more diverse segment of the population of problem drinkers [1,2]. Evidence supports the clinical effectiveness of a diversity of Web-based interventions varying from pure self-help to predominantly therapist-administered therapy [3-13], and it seems that the best results are achieved with interventions that use personalized feedback [3]. Despite these promising results, participants in Web-based interventions show great variation in how they use the interventions in terms of frequency and duration of visits, and they often do not complete treatment sessions or assessments [13-16]. However, Web-based intervention studies mainly focus on effectiveness, and less is known about the reasons for noncompletion and the specific components that improve adherence [15]. Although Web-based interventions have the potential of easy data collection, the study of attrition is still rare.

In his law of attrition, Eysenbach distinguished two processes of attrition: dropout attrition and nonusage attrition [15]. Dropout attrition refers to participants being lost to follow-up; they do not return to fill in follow-up questionnaires. Nonusage attrition refers to participants’ stopping to use the intervention, but still filling in questionnaires. Authors do not always describe dropout and nonusage attrition separately [4,8-10,17]. And if they do, then much variation is still possible within Eysenbach’s conceptualization, because of differences in treatment intervention and study design. Some studies, for example, only require filling out a single questionnaire in a shorter timeframe [4,10], while other studies require a wide range of questionnaires at a number of follow-up points [11,13]. Therefore, comparing attrition rates alone does not make sense. A clear description of the study characteristics, together with nonusage and dropout attrition, is necessary to interpret attrition data properly.

Usage and follow-up completion rates of Web-based alcohol interventions studies published to date range from 16.5% [18] to 92% [11]. In face-to-face addiction treatment, overall around 50% of patients terminate treatment before the intended period is over [19]. The great differences in attrition rates between Web-based interventions can be explained by differences in payment of incentives, follow-up periods, intensity and duration of the Web-based intervention, recruitment procedure, study population, and research environment (trial or open access). It seems to be the combination of factors that is responsible for the attrition rate rather than a single factor [4-6,8-13,18,20-23]. For example, Cunningham et al [11] found that 92% of participants completed baseline, 3-month, and 6-month follow-ups. This excellent follow-up completion rate might partly be explained by the incentive of a $20 check for each follow-up survey, but also by the way participants were recruited. Respondents from an ongoing telephone survey, who confirmed that they had home Internet access and were interested in a computerized program to check their drinking, were invited to participate in the study and therefore motivated respondents were recruited. Doumas and colleagues [10] also found a very good follow-up completion rate (88%) even without paying an incentive. However, their follow-up period was short, at 30 days, and the motivation for completing the study might have been greater for their population of mandated college students.

Attrition data have been mainly coming from trials. Compared with the dropout and nonusage attrition rates in effectiveness trials of Web-based interventions, attrition rates in open-access interventions are higher [14]. This might be due to the use of participant-retention strategies in trials and to the characteristics of trial participants (eg, motivated participants). The study of Linke et al [18], with a follow-up and usage completion rate of 16.5%, involved a cohort study with 10,000 users of a free, Web-based, 6-week intervention. They used a strict definition of attrition, as only registrants who completed the whole 6-week program and the final assessment were considered to be completers. In comparison, Cunningham et al [11] noted that, despite their excellent 92% follow-up completion rate at 6 months, 35 of the 92 participants in the intervention condition (38%) never accessed the intervention. Riper and colleagues investigated their self-help intervention (Drinking Less) in a randomized controlled trial (RCT) and an open-access sample. They reported a 54% follow-up completion rate for the 6-month follow-up in the RCT intervention group, and 45% of the baseline participants actually made use of the intervention [13]. In their open-access sample, they found a follow-up completion rate of 40.5% but 12% of participants never using the program, 60% using it once or a few times, and 28% using the intervention more than a few times [22]. The study examples above illustrate that providing access to an intervention does not guarantee that participants use it.

The high percentages of nonusage attrition lead to the question of whether Web-based alcohol treatment might work more effectively for some people than for others. Exploring the variables that make individuals more vulnerable to not completing treatment may help us to identify target groups and develop strategies to address the nonusage attrition problem. We examined three types of variables that were associated with nonusage or dropout attrition: sociodemographic variables, drinking behavior, and psychological variables. Those factors have been investigated in several online alcohol intervention studies. Although most studies found no differences in baseline variables between completers and noncompleters [5,8-11,13,17,21], other studies did find support for baseline differences. Sociodemographic variables found to be positively associated with intervention and follow-up completion were being female [12,18], married or living with a partner [18,22], and without children [18]. Riper and colleagues also found that follow-up noncompleters were more likely to be above the median age of 47 years [22]. Chiauzzi et al [6] found that study site (2 out of 5 universities) was a predictor of follow-up noncompletion. Regarding baseline drinking-behavior variables, intervention completers showed less risk of alcohol dependency and harm from alcohol [18], and consumed fewer units a week and per occasion than noncompleters [20]. Additionally, psychological predictor variables were found in two studies. Chiauzzi et al [6] found baseline stage of readiness for change (contemplation) to be a predictor of dropout attrition, and Postel et al [16] found that intervention completers had a higher baseline score on treatment readiness. It could be suggested that the results concerning the differences between completers and noncompleters are frequently ambiguous and are often found in only a single study. This might be the result of the differences in target groups and intervention characteristics. In line with this, Murray and colleagues [24] emphasized that it is important to adjust boosting strategies to the particular target population of the Web-based intervention. Whereas studies of online weight-loss programs, for example, have successfully boosted follow-up rates by using postal and telephone reminders for participants who did not respond to email reminders, Murray et al [24] received only 3% additional responses from their population of hazardous drinkers after an extensive additional follow-up using postal reminders and phone calls. It would be interesting to further investigate why such a strategy is working in one population but not in another one.

None of the Web-based alcohol intervention studies formally examined the reasons for noncompletion. Although most studies report the rates of nonusage or dropout attrition, they do not report the reasons for attrition. However, in our recently conducted RCT we examined the reasons for not completing treatment [16]. The Dutch Web-based treatment program (alcoholdebaas.nl) has been shown to be effective for problem drinkers in reducing their alcohol consumption and improving health status, yielding a large effect size at posttreatment [16]. The attrition rate in our Web-based treatment group (n = 42) was high at 54%. As we used a linear model for the treatment program with technically integrated assessment points, nonusage attrition automatically meant dropout attrition. Questionnaires could be sent to respondents only when all previous assignments were completed. Therefore, attrition was defined as not completing all 12 sessions of the Web-based intervention: 9 assignments and 3 assessments. We investigated reasons for noncompletion by sending an online questionnaire to all noncompleters. As described previously [16], the results showed that the main reasons for noncompletion in the Web-based treatment group were personal reasons unrelated to the Web-based treatment program, discomfort with the treatment protocol, and satisfaction with the positive results achieved to date. The present paper includes much more data regarding attrition in Web-based treatment for problem drinkers. We added the attrition data of the delayed control group and of a nontrial sample, and we conducted prediction analyses on pretreatment predictors of treatment completion. We also conducted qualitative analyses to get more insight into the reasons for dropout and participants’ suggestions for how to enhance the number of treatment completers.

The first aim of this study was to examine attrition prevalence and pretreatment predictors of attrition in a cohort of open-access users of the Web-based treatment program. The second aim was to further explore attrition data from our RCT. We investigated the prevalence of attrition, the reasons for noncompletion, pretreatment predictors of attrition, and participants’ suggestions for how to enhance treatment completion. Accordingly, the present study allowed us to compare the attrition data of both samples: a trial and an open-access group of users.

## Methods

### Study Design and Participants

The real-world sample consisted of all open-access users of the Web-based alcohol treatment program in 2009 (n = 885). The only inclusion criterion for open-access users was a minimum age of 18 years. All data entered by participants were stored in the Web-based application. We could identify who accessed the Web-based treatment program and who did not, the duration of participation for treatment completers, and the number of completed sessions in case of noncompletion. Participants who dropped out were not assessed about their situation at that time; because of the feasibility nature of the open-access study and the linear design it was not possible to send questionnaires to nonresponders through the application.

We conducted secondary analyses of our RCT: an open trial with participants randomly assigned to either the Web-based treatment group or to the waiting list control group [16]. The study protocol was approved by the independent medical ethics board METiGG (reference number NL20742.097.07) and registered at www.controlled-trials.com (ISRCTN39104853). In brief, we recruited Dutch-speaking problem drinkers in the general population aged ≥18 years. Problem drinking was defined as drinking currently at least 15 units (of 10 grams of ethanol) a week for women and 22 units a week for men. We excluded participants treated for problem drinking in the preceding year and participants with psychiatric treatment in the past 6 months or those currently with a psychiatric disorder. Of the problem drinkers screened (n=169), 156 were found to be eligible for the study, and they were randomly assigned to either the Web-based treatment group or to the waiting list control group. As the control group received the intervention immediately after the experimental group completed treatment, we merged the data from both groups for the present study. Participants received the e-therapy intervention free of charge. We did not provide any kind of incentive for study participation.

### Intervention

The Web-based alcohol treatment consisted of a structured, 2-part, online treatment program in which the participant and the therapist communicated asynchronously, via the Internet only. The method underlying the program was based on the principles of cognitive behavior therapy [25] and motivational interviewing [26]. Part 1 of the program consisted of 2 assessments and 4 assignments and focused on the analysis of the participants’ drinking habits. Part 2 focused on behavioral change and included 5 assignments and 1 final assessment. The average duration of the total treatment program was 3 months, with one or two therapist contacts per week and daily self-reporting of alcohol intake during the whole program. The 12 treatment sessions were identical for RCT and open-access users, except for the 3 assessments being more extensive for RCT participants.

### Outcome Measures

Participants’ pretreatment characteristics were derived online from the baseline self-report questionnaire, for RCT as well as for open-access participants. Weekly alcohol consumption was assessed by a 7-day retrospective drinking diary, including a question about atypical drinking [27]. Type and severity of substance dependence were assessed by the Substance Abuse Module of the Composite International Diagnostic Interview [28]. The 28-item General Health Questionnaire (GHQ-28) and the Maudsley Addiction Profile-Health Symptom Scale (MAP-HSS) were used to assess health status [29,30]. The 21-item Depression Anxiety Stress Scale (DASS-21) was used to measure the three related negative emotional states of depression, anxiety, and stress [31]. To measure the quality of life, the EQ-5D was used [32]. Initial treatment motivation was measured with the TCU Motivation for Treatment (MfT) scale [33], and participants’ readiness to change their drinking behavior was measured with the Dutch version of the Readiness to Change Questionnaire [34]. For open-access participants the questionnaires were less extensive, as the GHQ-28 and MAP-HSS were left out.

The outcome measure of the logistic regression analysis was completion of the Web-based alcohol treatment program; this was defined as completion of all 12 treatment sessions: 9 assignments and 3 assessments. Because of the linear design of the treatment program it was impossible for participants to skip parts of the intervention; therefore, the point at which they stopped using the program indicates exactly how much treatment participants received. In our study nonusage attrition automatically meant dropout attrition and we will therefore just use the term attrition.

In order to gain insight into the motives of participants to stop using the Web-based treatment program, noncompleters in the RCT group received an email with a link to an additional online questionnaire consisting mainly of open questions concerning their perception of the program, reasons for discontinuation, and suggestions to improve the intervention and enhance treatment completion. If participants did not complete this questionnaire, they were contacted by telephone to remind them to complete the questionnaire either online or alternatively by phone.

### Statistical Analysis

Chi-square and *t* tests were used to assess whether there were baseline differences between completers and noncompleters. Multivariate logistic regression analysis was performed with treatment completion as the dependent variable. Predictor variables with *P* < .10 in the univariate analyses were entered in a full multivariate model. Subsequently, nonsignificant variables were removed, one by one, until –2 log likelihood deteriorated significantly. Goodness of fit of the model was determined by the Hosmer-Lemeshow test, and the Nagelkerke R^2^ was used for the pseudo proportion of variance. Three regression analyses were performed concerning (1) the RCT sample, (2) the open-access sample including treatment readiness variable, and (3) the open-access sample without treatment readiness variable. Because treatment readiness was measured after part 1 in the open-access sample, we had a lot of missing data for this variable (n = 355). We therefore performed two regression analyses for the open-access sample, one including treatment readiness (and as a consequence only the noncompleters from part 2) and one without this variable (all noncompleters). The predictor variables for the RCT sample were age, gender, work, education level, baseline alcohol consumption, prior alcohol treatment, prior mental health treatment, readiness to change contemplation, and action score, DASS-21 total score, and the MfT questionnaire scores for desire for help and treatment readiness. For the open-access sample, the DASS-21 scores were not available and therefore left out of the regression analysis. All statistical tests were 2-sided, with *P* ≤ .05 considered to be significant, and performed using SPSS for Windows 17.0 (IBM Corporation, Somers, NY, USA).

Reasons for nonusage attrition were independently assessed by the first and third author (qualitative study). The agreement level between both authors was 87%, which was considered acceptable. If the two authors did not agree, the topic was discussed in order to reach agreement. Participants’ responses to open questions were analyzed using thematic analysis. The first author carefully searched through the data to identify and code all features concerning participants’ reasons for not completing the treatment program. After collating relevant data with each code, related patterns were combined into themes. After refining and defining the themes, a brief description of each theme was formulated related to the research questions of the study.

## Results

### Participant Characteristics

Of the 885 registrants for the open-access version in 2009, 105 never started using the Web-based alcohol treatment program by doing the first assignment, sending a message to their therapist, or logging into the daily alcohol diary. Of the 780 participants who started the open-access version, 54.0% (n = 421) were women, 49.6% (n = 387) had a higher education level, and 69.0% (n = 538) were employed. Age ranged from 20 to 78 years, with an average of 45.7 years (Table 1). A total of 689 participants reported alcohol dependence (88.3%), but many (n = 554, 71.0%) had never received professional help for their drinking problem. The mean weekly alcohol consumption was 42.7 standard units a week: 49.1 for men and 37.3 for women.

**Table 1:** Characteristics of participants in the randomized controlled trial (RCT) and open-access group

Variable		RCT participants (n = 144)		Open-access participants (n = 780)	
		n	%	n	%
Female		83	58	421	54.0
Higher education		84	58	387	49.6
Employed		117	81.3	538	69.0
**DSM-IV^a^ diagnosis**					
	Alcohol dependence	120	83.3	684	87.7
	Alcohol abuse	14	10	42	5
	No dependence or abuse	10	7	54	7
Prior treatment for alcohol abuse		22	15	226	29.0
Prior treatment mental health problems		72	50	455	58.3
**Problem drinking^b^**		144	100	689	88.3
		Mean	SD	Mean	SD
Age (years)		45.8	9.7	45.7	10.8
**Weekly alcohol consumption (standard units/week)**					
	Men	49.8	26.9	49.1	30.1
	Women	32.6	14.6	37.3	22.9
GHQ-28 score^c^		52.6	11.9	NA^d^	NA^d^
MAP-HSS score^e^		19.8	6.2	NA^d^	NA^d^
**DASS-21^f^**					
	Depression score	8.7	8.4	NA^d^	NA^d^
	Anxiety score	5.9	5.9	NA^d^	NA^d^
	Stress score	12.5	8.2	NA^d^	NA^d^
**RCQ^g^**					
	Precontemplation	12.1	1.3	12.3	1.6
	Contemplation	17.1	2.1	17.1	2.3
	Action	12.4	3.5	13.3	3.3
**MfT^h^**					
	Treatment Readiness	4.0	0.5	4.1	0.4
	Desire for Help	3.9	0.7	3.9	0.6

^a^ *Diagnostic and Statistical Manual of Mental Disorders*, 4th revision.

^b^ Drinking >21 (men) or >14 (women) mean units per week.

^c^ 28-item General Health Questionnaire.

^d^ Not applicable.

^e^ Maudsley Addiction Profile-Health Symptom Scale.

^f^ 21-item Depression Anxiety Stress Scale.

^g^ Readiness to Change Questionnaire.

^h^ TCU Motivation for Treatment scale.

**Figure 1:**
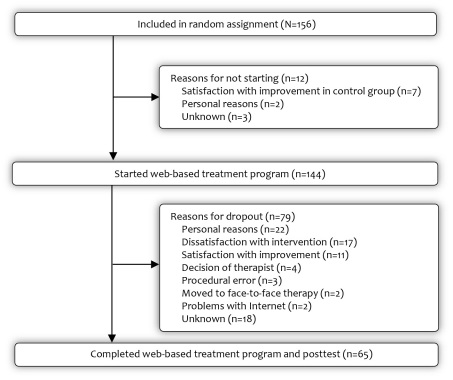
Flow of participants in the randomized controlled trial.

Figure 1 shows the participant flow of the total RCT sample (n = 144) along with reasons for not starting (n = 12). Pretreatment characteristics of the 144 RCT participants who started the Web-based treatment program are presented in Table 1. Of these participants, 58% (n = 83) were women, 58% (n = 84) had a higher education level, and 81.3% (n = 117) were employed. Ages ranged from 22 to 66 years, with an average of 45.8 years, and 120 participants reported dependence (83.3%). The majority (n = 122, 84.7%) had never received professional help for their drinking problem. The mean weekly alcohol consumption was 39.9 standard units a week: 49.8 for men and 32.6 for women.

### Attrition Pattern

Of the 780 open-access participants, 65.0% were noncompleters. Treatment completers (n = 273, 35.0%) completed all 12 treatment sessions and noncompleters (n = 507, 65.0%), an average of 4.8 (SD 3.1) sessions. Of the 144 RCT participants, 55% were noncompleters. Treatment completers (n = 65, 45%) completed all 12 treatment sessions and noncompleters (n = 79, 55%), an average of 4.8 (SD 3.1) sessions. The open-access and RCT group differed significantly in the percentage of treatment completers (χ^2^_1_ = 5.4; *P* = .02). Participants in the RCT sample were 1.29 (95% confidence interval [CI] 1.05–1.58) times more likely to complete treatment.

**Figure 2:**
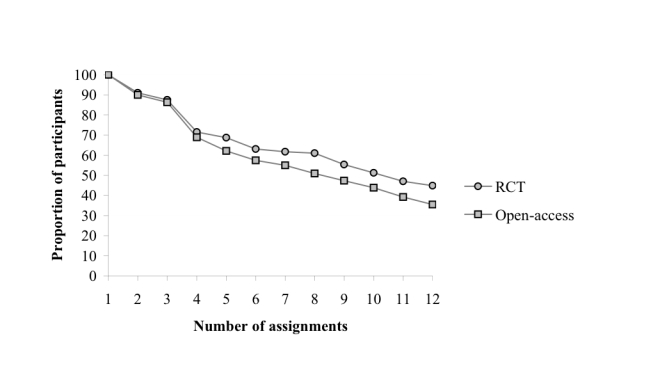
Attrition curve: proportion of participants by number of assignments in the randomized controlled trial (RCT) and open-access group.

Participants completed the sessions in the order that they were presented. The average duration of treatment to completion was 16.1 weeks in the RCT sample and 17.1 weeks in the open-access sample. Figure 2 shows the attrition curves of both groups. Participants dropped out during all stages of treatment. However, the biggest loss was found after the third session, possibly as a result of the daily drinking diary. In this session, participants were asked to register daily amounts of alcohol consumption for the whole treatment duration.

### Predictors of Treatment Completion

We found only one significant difference between completers and noncompleters in the RCT sample. The mean score on the Treatment Readiness subscale of the MfT was higher for completers (mean 4.13) than for noncompleters (mean 3.97), *t*_1,142_ = –2.00, *P* = .047. There were no other significant differences between the groups on any of the variables presented in Table 1. Logistic regression analysis revealed a statistically significant contribution of treatment readiness score. The regression equation showed a negative predicted value of 70% and a positive predictive value of 53%, with a cut-off probability of the model of 0.4. The Nagelkerke *R* *2* was .04, and the regression model showed sufficient goodness of fit (χ^2^_1_ = 10.7, *P* = .22). The area under the receiver operating characteristic (ROC) curve revealed a model discrimination value of 0.60 (95% CI 0.51–0.70). The odds ratio indicated that if the treatment readiness score increases by 1 point (range 1–5), the odds of completion increase with 2.1. A score of 3 gives a chance of completion of 27%, a score of 4 a chance of 44%, and a score of 5 a 63% chance.

We found seven significant differences between completers and noncompleters in the open-access sample: age, gender, education level, baseline alcohol consumption, prior mental health treatment, treatment readiness, and readiness to change action score. The differences are shown in Table 2. Multivariate logistic regression analysis with treatment readiness included (n = 425) revealed a statistically significant independent contribution of age, baseline alcohol consumption, and treatment readiness. Predicted probabilities of the model of x, y, and z led to a specificity of 89% with a sensitivity of 25%, a specificity of 84% with a sensitivity of 33%, and a specificity of 78% with a sensitivity of 40%, respectively. The Nagelkerke *R* *2* was .09, and the regression model showed sufficient goodness of fit (χ^2^_1_ = 11.7, *P* = .17). The area under the ROC curve revealed a discrimination of the model of 0.64 (95% CI 0.59–0.70). When the treatment readiness score increases by 1 point (range 1–5), the odds of completion increase 2.1-fold. If age increases by 5 years, the odds of completion increase 1.12-fold, and if baseline alcohol consumption increases by 10 standard units a week, the odds of completion decrease 0.87-fold.

**Table 2:** Differences between open-access completers and noncompleters

Variable	Completers (n = 273)		Noncompleters (n = 507)		Test result		
	n	%	n	%	χ^2^	df	*P* value
Female	170	62.3	251	48.5	11.6	1	<.001
Higher education	163	59.7	224	44.2	17.1	1	<.001
Prior mental health treatment	175	64.1	280	55.2	5.8	1	.02
	Mean	SD	Mean	SD	*t*	df	*P* value
Age (years)	47.8	10.4	44.5	10.9	–4.14	1,778	<.001
Baseline alcohol consumption (standard units/week)	37.4	24.0	45.6	28.2	4.05	1,778	<.001
MfT^a^ Treatment Readiness	4.1	0.4	4.0	0.4	–3.30	1,423	.001
RCQ^b^ action score	13.8	3.3	13.0	3.3	–3.43	1,778	<.001

^a^ TCU Motivation for Treatment scale.

^b^ Readiness to Change Questionnaire.

Multivariate logistic regression analysis without treatment readiness (n = 780) revealed a statistically significant contribution of age, gender, education level, baseline alcohol consumption, and readiness to change action score. Predicted probabilities of the model of x, y, and z led to a specificity of 85% with a sensitivity of 25%, a specificity of 80% with a sensitivity of 35%, and a specificity of 75% with a sensitivity of 43%, respectively. The Nagelkerke *R* *2* was .10, and the regression model showed sufficient goodness of fit (χ^2^_1_ = 7.1, *P* = .53). The area under the ROC curve revealed a discrimination of the model of 0.63 (95% CI 0.59–0.67). The odds of treatment completion was 1.70-fold increased for women compared with men, and 1.79-fold increased for people with higher education compared with less-educated people. The odds ratios further indicated that if age increases by 5 years, the odds of completion increase 1.13-fold, and if baseline alcohol consumption increases by 10 standard units, the odds of completion decrease 0.93-fold. If the readiness to change action score increases by 1 point (range 4–20), the odds of completion increase 2.1-fold.

### Early Versus Late Noncompleters

We divided noncompleters into early and late noncompleters to determine whether the two groups differed. We considered noncompleters who completed a maximum of 3 assignments to be early noncompleters and those who completed at least 4 assignments to be late noncompleters. We found no differences between both groups in the RCT sample (n = 144). However, in the open-access sample (n = 780) we found that, compared with those who completed fewer assignments, more noncompleters who completed at least 4 assignments had a high level of education (128/221, 57.9% vs 93/221, 42%, χ^2^_1_ = 6.1, *P* = .01), had received prior mental health treatment (162/276, 58.7% vs 114/276, 41.3%, χ^2^_1_ = 12.0, *P* < .001), and had a lower baseline alcohol consumption (43.2 vs 48.3 standard units a week, *t*_501_ = 2.01, *P* = .045).

### Reasons for Noncompletion

Figure 1 shows the reasons for noncompletion (n = 79). Self-reported reasons for not completing treatment were collected only in the RCT sample, and were obtained from 61 of 79 participants (77%). We were not able to contact 18 participants because of nonresponse or an invalid phone number. The most common reason for not completing treatment consisted of personal reasons unrelated to the Web-based intervention (n = 22), followed by dissatisfaction with the intervention (n = 17), and satisfaction with the improvement in their condition (n = 11). On four occasions the therapist decided to terminate the treatment, because of insufficient response or information (n = 3) or due to an inability to set a realistic drinking goal (n = 1). Unfortunately, in three cases we had procedural problems during the trial, and those participants could not continue. Additionally, 2 participants moved on to face-to-face treatment and 2 participants experienced problems with the Internet during treatment participation.

#### Personal Reasons

A diversity of personal reasons were given as reason for noncompletion (n = 22), including being too busy with work, a seriously ill family member or bereavement, other priorities, a hospitalization, no Internet access, or moving house.

#### Dissatisfaction With Intervention

Participants who identified the Web-based alcohol intervention itself as a reason for discontinuation (n = 17) most commonly indicated that the program was too time consuming or too demanding. Some participants reported that the program could not meet their personal needs.

#### Improvement in Condition

Several participants reported that they no longer felt the need to continue the program, because of the progress they made (n = 11). They gained from the intervention what they needed and felt in control of their drinking behavior.

#### Other Reasons

For 2 participants the Web-based treatment program was only the first step in working on behavioral change, and they continued treatment in a face-to-face setting. Of the persons whose formal reason for dropout is unknown (n = 18), the messages in their personal records provide some information. Participants mentioned several times that working on their alcohol problem was quite confrontational and overwhelmed them too much. Some participants also reported more or less lack of motivation.

### Suggestions to Enhance Treatment Completion

Several RCT participants gave suggestions as how to improve the Web-based treatment program. One of the suggestions was sending an email message to participants to notify them that they had received a new message or assignment from their therapist. This it was felt would act as a reminder and prevent unnecessary logging into the application. Another suggestion was to allow more flexibility in the treatment protocol, with the possibility of skipping sessions when required—for example, the possibility to start immediately with the goal-setting assignment or no longer mandating daily registration. In its current form it was not possible to move on to the next assignment without completing the previous one. Some participants also mentioned the need for additional contact: the choice to contact their therapist by phone or face-to-face and the chance to get in touch with fellow participants, with the suggestion to link each participant to his or her own buddy. Some participants made suggestions for improving the usability of the Web-based treatment program, including the speed of the intervention, layout characteristics, and button functions.

## Discussion

### Main Findings

The aim of this study was to explore the attrition data of an open-access and an RCT sample of a Web-based treatment program for problem drinkers. The study demonstrated high prevalence of attrition in both samples, with 10% less treatment completers in the open-access sample. Participants’ readiness for treatment, gender, education level, age, baseline alcohol consumption, and readiness to change score were shown to predict treatment completion. The key reasons for noncompletion were personal reasons, dissatisfaction with the intervention, and satisfaction with their own improvement. The main suggestions for boosting strategies involved email notification and more flexibility in the intervention.

### Attrition

Attrition was high in both samples. Although our attrition rates of 65% in the open-access sample and 55% in the RCT sample are in line with those found in other Web-based alcohol intervention studies [12,13,22], the majority of alcohol intervention studies found lower attrition rates [4-6,8-11,17,21]. However, comparing attrition rates alone does not make sense. A clear description of the study characteristics together with nonusage and follow-up attrition is necessary to interpret attrition data properly. Our attrition rates need to be seen in the light of a strict definition of treatment completion including assessment completion, active usage of the intervention, a high intensity of the treatment program, and paying no incentive to participants. In comparison, Linke et al [18] used a similar definition of attrition in their cohort sample of the brief intervention Down Your Drink and found a completion rate of 16.5%. To the best of our knowledge, no online alcohol intervention studies have been published concerning comparable guided treatment with intensive therapist contact. We therefore can only compare our attrition rates with those of more or less intensive online alcohol self-help interventions. Although there is some evidence from computer-aided psychological treatment programs that participants receiving extra therapist contact (eg, phone support) drop out less often, no studies have explored the influence of therapist contact on dropout from Web-based treatments for psychological disorders [35]. Further investigation of the impact of therapist contact on attrition from online alcohol interventions is needed.

The variety of nonusage and dropout attrition rates in Web-based alcohol interventions is relatively similar to that found in Web-based treatments for psychological disorders, ranging from 2% to 83% [35]. A higher number of noncompleters in our open-access sample is consistent with earlier findings [14]. The fact that RCT participants were 1.5 times more likely to complete treatment might be the result of a selection bias, because of the prescreening of trial participants and the exclusion criteria. It leads to the suggestion that it might be wise to always link some kind of research to a Web-based intervention and to emphasize the importance of it at the start. Realizing that you are cooperating in a research project, for example to improve the intervention, can perhaps be inspiring. We acknowledge that it is important to find a good balance between what is needed for attrition purposes and what is considered to be ethically appropriate. Finding the right tone seems to be important. Further research needs to investigate whether this strategy will be effective in reducing the number of dropouts, and whether this works for participants and for therapists. What is the impact of this for participants? Do therapists change the treatment or the communication with participants if they know that the data will be used for research purposes? Are therapists extra motivated to increase adherence to the treatment protocol?

In both study samples, the pattern of nonusage attrition was steady throughout the intervention period. This means that both groups showed the same trend of attrition; at each treatment session participants dropped out. The number of dropouts gradually decreased, regardless of whether participants participated in the RCT or in the open-access intervention. Although the gradual decrease is in contrast with the suggestion of Eysenbach [15] that, in the final stage of an intervention, a hardcore group of users remain who will continue using the intervention, it is identical to the attrition pattern found by Neve and colleagues [36] in their 12-week, Web-based weight-loss program.

The percentage of dropouts seems to be the highest after session 3, concerning the daily drinking diary assignment. A possible explanation might be the intensity of this assignment, as participants have to register their alcohol consumption every day. This might be quite confrontational and participants might also feel uncomfortable or annoyed by daily registration of their drinking amount.

The differences we found between early and late noncompleters prove that noncompleters who completed at least 4 assignments were more similar to treatment completers than they were to those who completed fewer than 4 assignments.

### Predictors of Completion

The only statistically significant predictor of treatment completion in the RCT sample was a higher treatment readiness score, measured by the Treatment Readiness subscale of the TCU MfT questionnaire. In the open-access sample, higher treatment readiness also was a significant predictor, as were higher age and lower baseline alcohol consumption when the treatment readiness variable was included (n = 425). In the open-access sample without the treatment readiness variable (n = 780), the statistically significant predictors were higher age, female gender, higher education level, lower baseline alcohol consumption, and higher readiness to change action score. Other factors were found to have no predictive value.

Based on our different findings in the three subsamples and in line with an analysis of the literature by Melville and colleagues [35], we have to conclude that the current evidence for predictors of attrition is ambiguous. Two other Web-based alcohol intervention studies previously found that study completers consumed less alcohol at baseline [20,22]. Earlier studies by Bewick et al [12] and Lange et al [37] also found that more men than women were noncompleters, although Riper et al [22] did not find a significant association between gender and dropout. Male gender was also found to be associated with noncompliance in face-to-face addiction treatment [19]. A higher education level as a predictor of completion was not confirmed by three studies that explored the influence of education level on dropout from Web-based interventions; they did not find a significant association [22,37,38]. However, the association between compliance and higher education level was confirmed in face-to-face addiction treatment [19]. With regard to age, previous evidence was contrary to our findings. Riper et al [22] found that noncompleters were more likely to be above the median age of 47 years, whereas we found that noncompleters were younger than completers. Previous Web-based intervention studies also did not confirm the differences in treatment readiness between completers and noncompleters and found no predictive value for readiness to change [12]. But lower intention to comply with treatment and weaker initial treatment motivation were found to be associated with noncompliance in face-to-face addiction treatment [19]. The relationship between the baseline variables and dropout might also be mediated by other variables. Older participants or more highly educated participants might, for example, use the Internet in a different manner from younger or less-educated participants. Women probably experience more support from their relatives, which might stimulate continuation of treatment. And participants with lower baseline alcohol consumption may have more confidence in their own effectiveness. It would be interesting to further investigate the relationship between baseline variables and dropout. Overall, our findings also raise the question of how useful this kind of prediction research is. Because of the considerable variation in findings, we would on the one hand suggest that further research is needed to confirm whether the same predictors exist in different Web-based alcohol interventions, but on the other hand we would also suggest not focusing too much on baseline predictors of online treatment completion. It might be more effective to focus on the therapist side and the effects of boosting strategies in online interventions. The clinical implications of this study can therefore only be given with caution. It would be interesting to investigate whether increasing treatment readiness and readiness to change immediately from the start of treatment would decrease the number of noncompleters. Additionally it might be interesting to find out whether it matters how fast participants reduce their alcohol consumption or become abstinent after the start of the treatment program. Another question could be whether the pace at which participants experience a positive relationship with their therapist also has an effect on treatment completion.

### Reasons for Noncompletion

In addition to the quantitative data of the RCT and open-access sample, the qualitative data provided more insight into the reasons for noncompletion and the possibilities to reduce potential loss. The present more extensive findings confirm the earlier findings on dropout from our RCT study and, as discussed before [16], most reasons for noncompletion are in line with the potential factors for attrition as described in the law of attrition by Eysenbach [15], except for improvement in condition. Some participants significantly improved after just a few treatment sessions, and they were convinced that no additional sessions were needed. This confirms Christensen and Mackinnon’s statement that low usage and dropout do not necessarily coincide with failure [39]. Participants who do not complete the treatment program or follow-up assessments may still derive much benefit from the Web-based intervention. Continuous and frequent measurement, such as with diary surveys, can provide the necessary data [40]. Although a disadvantage of diary surveys is that the respondents themselves are responsible for completion, a Web-based intervention has the potential to easily prompt users by automatically sending reminders, motivational messages, or incentives. We also suggest investing in easy referral from Web-based treatment to face-to-face treatment with the possibility of integrated treatment (Web-based and face-to-face). Participants as well as their therapists expressed interest in this kind of integrated care. Professionals at the International Network on Brief Interventions for Alcohol Problems conference also expressed interest in this possibility [41].

### Boosting Strategies

Boosting strategies are desirable to maximize the number of treatment completers in trial settings as well as in open-access interventions. Participants themselves suggested sending email reminders as an additional supportive resource. The use of push reminders, such as phone calls, postcards, and email messages, previously has shown improved treatment completion rates [42,43]. Although participants already received therapists’ messages in the Web-based application, they preferred receiving reminders in their private email account in order to be constantly reminded of their participation and to prevent unnecessary logging into the application. Participants also suggested more flexibility in the Web-based treatment program. The most frequently mentioned response was that daily alcohol registration was somewhat annoying to participants. This might explain the more pronounced loss of participants (16%–17%) after the third session, as this assignment requested starting with daily alcohol registration. Another suggestion was to better adapt the pace of the treatment to the needs of the individual participant and not being too rigid in terms of the fixed treatment duration. Interestingly, none of the participants suggested incentives as a useful boosting strategy, possibly because they thought this was embarrassing to suggest. Contingency management interventions have been shown to increase desired behavior by offering valuable reinforcements contingent on behavioral change [44]. It would be an interesting direction for future research to apply the contingency management principles in Internet interventions and to investigate their effectiveness.

### Methodological Considerations

This study has several limitations that are important to acknowledge. Due to the technical structure of our intervention, noncompletion included not just stopping using the intervention but also no longer receiving posttest and follow-up assessments. The therapists and participants could not move on to the next assignment or questionnaire without completing the previous one. We chose this linear model because of the protocolled treatment and the preference for completing treatment steps in strict order, to ensure best quality and that the questionnaires would be completed. However, a consequence that we have not sufficiently taken into account is that nonusage attrition also meant study attrition and that we unfortunately never obtained a lot of data from noncompleters. This is definitely not desirable and needs to be changed in future studies. One of the consequences is that we did not have data available to compare treatment outcome of completers versus noncompleters. Although our qualitative data indicated that completers had better treatment results, this assumption can be confirmed only with quantitative data. As far as we know, no previous online alcohol intervention study has investigated the difference in treatment outcome between completers and noncompleters. We therefore recommend investigating the impact of compliance on treatment outcome in future studies.

We also decided not to use push factors in our RCT to keep the trial setting as natural as possible. However, it is possible that, if we had used push factors, we could have raised the response rate to generate a more complete dataset.

Another limitation is that only baseline characteristics were considered as potential predictors of treatment completion. It is possible that other factors such as forum use or the therapeutic relationship also influenced attrition rate. However, we aimed to determine at baseline which participants would complete the whole treatment program. We were also limited to the baseline characteristics we measured and therefore not able to include some of the variables previously found to have predictive value.

Both study samples consisted largely of adults in their mid-40s. This can partly be explained because our samples consisted of problem drinkers from the general public. And although we previously found that the average age of face-to-face clients was slightly lower, face-to-face clients also have a mean age of around 43 years [1,45]. It often takes a long time before people experience excessive alcohol consumption as a problem. The physical and psychological damage will only be felt over time. People in their mid-40s often take responsibility for their own health and are looking for a healthier lifestyle, including drinking less. Web-based treatment is a pleasant option for them, because of the privacy and easy access to online help. Although they are an important target group for our intervention, it remains a challenge to reach younger and older problem drinkers via the Internet as well. Future research should focus on how these groups can be reached.

### Future Directions and Implications

Nowadays, the challenge of Web-based alcohol treatment programs no longer seems to be their effectiveness but keeping participants involved until the end of the treatment program. Our study provided some points that therapists might focus on, including helping participants to be ready for treatment and for change. We should also investigate the effect of starting immediately with reduction of alcohol consumption. Boosting strategies such as email notification and more flexibility in the intervention might also help to improve adherence. Further research should investigate whether those changes lead to decreased attrition rates in Web-based interventions. If we can succeed in improving attrition rates, we assume that the success of Web-based alcohol interventions will further improve and, as a consequence, they will have a greater public health impact.

## Abbreviations

CI: confidence interval
DASS-21: Depression Anxiety Stress Scale
DSM-IV: Diagnostic and Statistical Manual of Mental Disorders, 4th revision
GHQ-28: 28-item General Health Questionnaire
MAP-HSS: Maudsley Addiction Profile-Health Symptom Scale
MfT: TCU Motivation for Treatment scale
RCQ: Readiness to Change Questionnaire
RCT: randomized controlled trial
ROC: receiver operating characteristic
